# Q-HAM: a multicenter upfront randomized phase II trial of quizartinib and high-dose Ara-C plus mitoxantrone in relapsed/refractory AML with FLT3-ITD

**DOI:** 10.1186/s13063-023-07421-x

**Published:** 2023-09-15

**Authors:** Sonia Jaramillo, Lucian Le Cornet, Markus Kratzmann, Johannes Krisam, Martin Görner, Mathias Hänel, Christoph Röllig, Maxi Wass, Sebastian Scholl, Mark Ringhoffer, Alexander Reichart, Björn Steffen, Sabine Kayser, Jan-Henrik Mikesch, Kerstin Schaefer-Eckart, Jörg Schubert, Thomas Geer, Sonja Martin, Meinhard Kieser, Tim Sauer, Katharina Kriegsmann, Michael Hundemer, Hubert Serve, Martin Bornhäuser, Carsten Müller-Tidow, Richard F. Schlenk

**Affiliations:** 1 Department of Internal Medicine V, Heidelberg University Hospital, Heidelberg, Germany; 2 NCT-Trial Center, National Center of Tumor Diseases, Heidelberg University Hospital and German Cancer Research Center, Heidelberg, Germany; 3 Institute of Medical Biometry, University of Heidelberg, Heidelberg, Germany; 4 Department of Hematology, Oncology and Palliative Medicine, Community Hospital Bielefeld, Bielefeld, Germany; 5 Department of Medicine III, Hospital Chemnitz gGmbH, Chemnitz, Germany; 6 Department of Medicine and Polyclinic I, TU Dresden University Hospital, Dresden, Germany; 7 Department of Medicine IV, Halle (Saale) University Hospital, Halle, Germany; 8 Department of Medicine II, Jena University Hospital, Jena, Germany; 9 Department of Medicine, III, Hospital Karlsruhe, Karlsruhe, Germany; 10 Department of Hematology, Oncology and Palliative Medicine, Hospital Winnenden, Winnenden, Germany; 11 Department of Medicine II, Frankfurt University Hospital, Frankfurt, Germany; 12 Department of Medicine I – Hematology and Cell Therapy, Leipzig University Hospital, Leipzig, Germany; 13 Department of Medicine A, Münster University Hospital, Münster, Germany; 14 Department of Inner Medicine V, North Hospital Nürnberg, Nürnberg, Germany; 15 Department of Inner Medicine II, Elbland Hospital Riesa, Riesa, Germany; 16 Department of Medicine II, Diaconal Hospital Schwäbisch-Hall, Schwäbisch Hall, Germany; 17 Department of Hematology, Oncology and Palliative Medicine, Robert-Bosch Hospital, Stuttgart, Germany

**Keywords:** Quizartinib, Relapse, Refractory, Acute myeloid leukemia, Measurable residual disease, Matched threshold crossing approach

## Abstract

**Background:**

About 50% of older patients with acute myeloid leukemia (AML) fail to attain complete remission (CR) following cytarabine plus anthracycline-based induction therapy. Salvage chemotherapy regimens are based on high-dose cytarabine (HiDAC), which is frequently combined with mitoxantrone (HAM regimen). However, CR rates remain low, with less than one-third of the patients achieving a CR. *FLT3*-ITD has consistently been identified as an unfavorable molecular marker in both relapsed and refractory (r/r)-AML. One-quarter of patients who received midostaurin are refractory to induction therapy and relapse rate at 2 years exceeds 40%. The oral second-generation bis-aryl urea tyrosine kinase inhibitor quizartinib is a very selective *FLT3* inhibitor, has a high capacity for sustained *FLT3* inhibition, and has an acceptable toxicity profile.

**Methods:**

In this multicenter, upfront randomized phase II trial, all patients receive quizartinib combined with HAM (cytarabine 3g/m^2^ bidaily day one to day three, mitoxantrone 10mg/m^2^ days two and three) during salvage therapy. Efficacy is assessed by comparison to historical controls based on the matched threshold crossing approach with achievement of CR, complete remission with incomplete hematologic recovery (CRi), or complete remission with partial recovery of peripheral blood counts (CRh) as primary endpoint. During consolidation therapy (chemotherapy and allogeneic hematopoietic cell transplantation), patients receive either prophylactic quizartinib therapy or measurable residual disease (MRD)-triggered preemptive continuation therapy with quizartinib according to up-front randomization.

The matched threshold crossing approach is a novel study-design to enhance the classic single-arm trial design by including matched historical controls from previous clinical studies. It overcomes common disadvantages of single-armed and small randomized studies, since the expected outcome of the observed study population can be adjusted based on the matched controls with a comparable distribution of known prognostic and predictive factors. Furthermore, balanced treatment groups lead to stable statistical models. However, one of the limitations of our study is the inability to adjust for unobserved or unknown confounders.

Addressing the primary endpoint, CR/CRi/CRh after salvage therapy, the maximal sample size of 80 patients is assessed generating a desirable power of the used adaptive design, assuming a logistic regression is performed at a one-sided significance level *α*=0.05, the aspired power is 0.8, and the number of matching partners per intervention patient is at least 1. After enrolling 20 patients, the trial sample size will be recalculated in an interim analysis based on a conditional power argument.

**Conclusion:**

Currently, there is no commonly accepted standard for salvage chemotherapy treatment. The objective of the salvage therapy is to reduce leukemic burden, achieve the best possible remission, and perform a hemopoietic stem-cell transplantation. Thus, in patients with *FLT3*-ITD mutation, the comparison of quizartinib with intensive salvage therapy versus chemotherapy alone appears as a logical consequence in terms of efficacy and safety.

**Ethics and dissemination:**

Ethical approval and approvals from the local and federal competent authorities were granted. Trial results will be reported via peer-reviewed journals and presented at conferences and scientific meetings.

**Trial registration:**

ClinicalTrials.gov NCT03989713; EudraCT Number: 2018-002675-17.

**Supplementary Information:**

The online version contains supplementary material available at 10.1186/s13063-023-07421-x.

## Roles and responsibilities

**Table:** 

Coordinating Investigator Prof. Dr. Richard F. Schlenk Trial Center Im Neuenheimer Feld 130/3 D-69120 Heidelberg	Joint Coordinating Investigator Prof. Dr. Carsten Müller-Tidow Department of Internal Medicine V Heidelberg University Hospital Im Neuenheimer Feld 410 D-69120 Heidelberg
Sponsor Ruprecht-Karls-University of Heidelberg Medical Faculty represented in law by Heidelberg University Hospital and its Commercial Managing Director Mrs. Katrin Erk, Im Neuenheimer Feld 672 D-69120 Heidelberg	Medical Coordinator Dr. Sonia Jaramillo Department of Internal Medicine V University Hospital Heidelberg Im Neuenheimer Feld 410 D-69120 Heidelberg
Biostatistician Dr. Johannes Krisam Institute of Medical Biometry and Informatics Heidelberg University Hospital Im Neuenheimer Feld 130.3 D-69120 Heidelberg	Supervising Statistician Prof. Dr. Meinhard Kieser Institute of Medical Biometry and Informatics Heidelberg University Hospital Im Neuenheimer Feld 130.3 D-69120 Heidelberg
Trial Coordination Dr. Lucian Le Cornet Dr. Markus Kratzmann NCT Trial Center Im Neuenheimer Feld 130/3 D-69120 Heidelberg	Data Management Frau Olga Eissymont Institute of Medical Biometry and Informatics Heidelberg University Hospital Im Neuenheimer Feld 130.3 D-69120 Heidelberg
Monitoring KKS Heidelberg Heidelberg University Hospital Im Neuenheimer Feld 130/3, D-69120 Heidelberg	Pharmacovigilance KKS Heidelberg Heidelberg University Hospital Im Neuenheimer Feld 130/3, D-69120 Heidelberg
Central molecular genetics Central MRD Laboratory Dr. Katharina Kriegsmann PD Dr. med. Michael Hundemer Hematological diagnostic laboratory Department of Internal Medicine V Im Neuenheimer Feld 410 / Room 01.136 D-69120 Heidelberg	Quality of Life / Patient-Reported Outcomes Prof. Dr. Karen Steindorf Division of Physical Activity, Prevention and Cancer German Cancer Research Center Im Neuenheimer Feld 460 D-69120 Heidelberg
Central reference pathology Prof. Dr. Claudia Wickenhauser Universitätsklinikum Halle (Saale) Institut für Pathologie Magdeburger Straße 14 06112 Halle/Saale	

## Background

Intensive chemotherapy consisting of induction and consolidation therapy is given with curative intent in most of the patients with acute myeloid leukemia (AML) except for old and frail patients [1–3]. Despite intensive therapy, the long-term outcome of AML patients remains poor, with less than 30% of patients achieving long-lasting remission or even cure [1, 2]. This poor outcome is mainly due to refractoriness to induction chemotherapy and relapse during and after completion of intensive induction and consolidation therapy. About 20–30% of AML patients under the age of 60 years and about 50% of older patients fail to attain complete remission (CR) following cytarabine plus anthracycline-based standard induction therapy [4–6]. Furthermore, patients having achieved CR are at a high risk of relapse, particularly within the first 2 years after completion of chemotherapy [7].

Allogeneic hematopoietic cell transplantation (allo-HCT) is currently the only treatment strategy to offer the prospect of a cure in relapsed/refractory (r/r-) AML. The outcome of allo-HCT is heavily influenced by the remission state before allo-HCT [8–12], and according to recent publications, measurable residual disease (MRD) positivity in both peripheral blood and bone marrow (BM) was consistently revealed to be a poor prognosis factor regarding relapse rate [8, 9]. Salvage chemotherapy regimens are administered in r/r-AML to induce CR before allo-HCT. Typically, these salvage regimens are based on high-dose cytarabine (HiDAC), which is frequently combined with either mitoxantrone (HAM regimen) or fludarabine plus idarubicin (idaFLA regimen) [10]. However, there is still no commonly accepted standard salvage regime, and overall CR rates are low [10].

Internal tandem duplications of the FMS-related tyrosine kinase 3 (*FLT3*-ITD) have consistently been identified as an unfavorable molecular marker in both relapsed and refractory AML [7, 10]. Furthermore, patients with refractory disease exhibiting a *FLT3*-ITD showed, despite an allo-HCT, a hazard ratio (HR) of 1.5 (95% CI 1.2–1.7) higher probability of death [6].

Midostaurin is currently the only tyrosine kinase inhibitor (TKI) that has demonstrated superior results compared to standard intensive therapy in younger *FLT3*-mutated newly diagnosed AML patients for all survival endpoints, including overall survival [11]. Midostaurin is now approved for the treatment of AML in newly diagnosed patients with an activating *FLT3*-mutation in combination with intensive induction, consolidation including allo-HCT, and maintenance therapy in the EU. Furthermore, based on a phase II follow-up study of the RATIFY trial, the approval was extended to older patients aged between 60 and 70 years [12]. However still, roughly one-quarter of patients in the midostaurin arm of the study were refractory to induction therapy and relapse rate at 2 years exceeded 40% [11]. Thus, new treatment options are urgently needed, particularly for r/r-AML with *FLT3*-ITD.

The oral second-generation bis-aryl urea inhibitor quizartinib is a very selective* FLT3* inhibitor, has a high capacity for sustained *FLT3* inhibition, and an acceptable toxicity profile [13]. In a phase II study (*n*=333), quizartinib demonstrated particular efficacy in patients with *FLT3*-ITD mutations (*n*=248), who were relapsed or refractory to 2nd-line, salvage chemotherapy, or relapsed after allo-HCT [14]. The response to single-agent quizartinib in *FLT3*-ITD-positive patients was overall 50.4% (125/248), 56% in patients with r/r-AML within the first-line therapy first year, and 46% after allo-HCT. A subsequent phase II study recruited 76 patients with *FLT3*-ITD mutations with r/r-AML after either one second-line therapy or allo-HCT. Patients were randomized to single-agent quizartinib 30mg/day (Group A) or 60mg/day (Group B) given as single agent orally during 28-day continuous treatment cycles, until relapse, intolerance, or proceeding to allo-HCT. The response rate was 61% in Group A and 71% in Group B. In addition, 32% of patients in Group A and 42% in Group B could be successfully bridged to allo-HCT [15]. Recently, first results of the randomized study in relapsed (with a duration of first CR of 6 months or less)/refractory AML have been reported comparing single-agent quizartinib (*n*=245) to investigator’s choice (*n*=122) [16]. In this setting, single-agent quizartinib improved OS significantly (HR 0.76, 95% CI 0.58–0.98; stratified log-rank test, 1-sided *P*=0.0177). Median OS was 27 weeks (95% CI 23.1–31.3) and 20.4 weeks (95% CI 17.3–23.7) for patients treated with quizartinib and investigator’s choice, respectively. At 1 year, the estimated OS probability was 27% for the quizartinib and 20% for investigator’s choice. Although quizartinib was superior compared to investigator’s choice, results may improve when quizartinib is combined with intensive chemotherapy.

Feasibility of combination therapy with quizartinib and chemotherapy has been shown in several phase II trials [17, 18] inducing a double-blinded, randomized phase III study of quizartinib with induction and consolidation chemotherapy and as maintenance in patients with newly diagnosed *FLT3*-ITD AML (ClincalTrials.gov identifier: NCT02668653) [19].

Regarding dosage and schedule, in the first-in-human phase I study, CP0001, quizartinib was administered with intermittent dosing (14 days on drug followed by 14 days rest) from 12 to 450mg and continuous dosing at 200mg and 300mg for 28 days in 76 patients with r/r-AML, regardless of *FLT3*-ITD mutation status [13]. Plasma taken from subjects and assayed in an in vitro plasma inhibitory assay (PIA) showed rapid and durable inhibition of *FLT3* phosphorylation as early as 2 h after the first dose. The overall response rate was 53% in *FLT3*-ITD mutated and 14% in *FLT3*-ITD unmutated patients [13].

The response rate observed in Study CP0001 was confirmed in the phase II study, AC220-002, of single-agent quizartinib in r/r AML. In this phase II study, a total of 333 patients were enrolled in 2 cohorts; Cohort 1 included patients 60 years or older who were relapsed or refractory to the first line of therapy, and Cohort 2 included patients 18 years or older who were relapsed or refractory to salvage therapy or relapsed after allo-HCT. In Cohort 1, the composite complete remission (CRc) rate was 57% in *FLT3*-ITD mutated patients with a median survival of 25.3 weeks [20, 21]. Cohort 2 showed a CRc rate of 46% in *FLT3*-ITD mutated patients with a median survival of 24.0 weeks [20]. Notably, 35% of Cohort 2 *FLT3-*ITD mutated patients were bridged to allo-HCT [22].

The maximum tolerated dose (MTD) determined in the phase I study, CP0001, was 200mg continuous daily dosing [13]. However, in the phase II study AC220-002, 35% of patients experienced grade 3 QT prolongation at the 200mg dose, and therefore the dose was reduced. A single case of grade 4 QT prolongation, Torsades de Pointes, was reported in the AC220-002 Study in a patient with pneumonia, atrial fibrillation, taking concomitant medications known to cause QT prolongation [23]. No deaths related to QT prolongation have been reported [20].

The phase IIb 2689-CL-2004 Study was subsequently conducted. A total of 76 patients with *FLT3-*ITD-mutated AML were randomized to 60mg or 30mg quizartinib daily to examine the efficacy and toxicity at these lower doses. Both males and females were randomized to each dose. The study showed that the CRc rate was similar for both doses and to the rate observed in the earlier AC220-002 Study [24].

Common adverse events (AEs) observed in the phase I and II studies included gastrointestinal disorders (nausea, diarrhea, and vomiting), hematologic disorders (anemia, neutropenia, and thrombocytopenia), febrile neutropenia, fatigue, and QT prolongation.

Although hematologic toxicity is associated with the underlying disease, safety reports from Study AC220-002 in AML indicate delayed recovery or continued suppression of absolute neutrophil counts (ANC) and platelets as a consequence of continued treatment with quizartinib [20].

Based on phase I and phase II studies’ results, it appears reasonable and clinically feasible to combine salvage therapy with quizartinib in patients with r/r- *FLT3*-ITD mutated AML. This manuscript describes the rationale, design, and dosing details of the Q-HAM study (clinicaltrials.gov identifier NCT03989713; EudraCT No 2018-002675-17), a phase II study comparing quizartinib adjunct to salvage therapy to historical controls based on the matched threshold crossing approach.

The primary objectives of the study are (i) to assess the clinical efficacy of quizartinib in combination with high-dose cytarabine and mitoxantrone and (ii) to assess the clinical efficacy of MRD-triggered and preemptive quizartinib continuation therapy in patients with r/r- *FLT3*-ITD mutated AML.

## Materials and methods

### Design

**Fig. 1:**
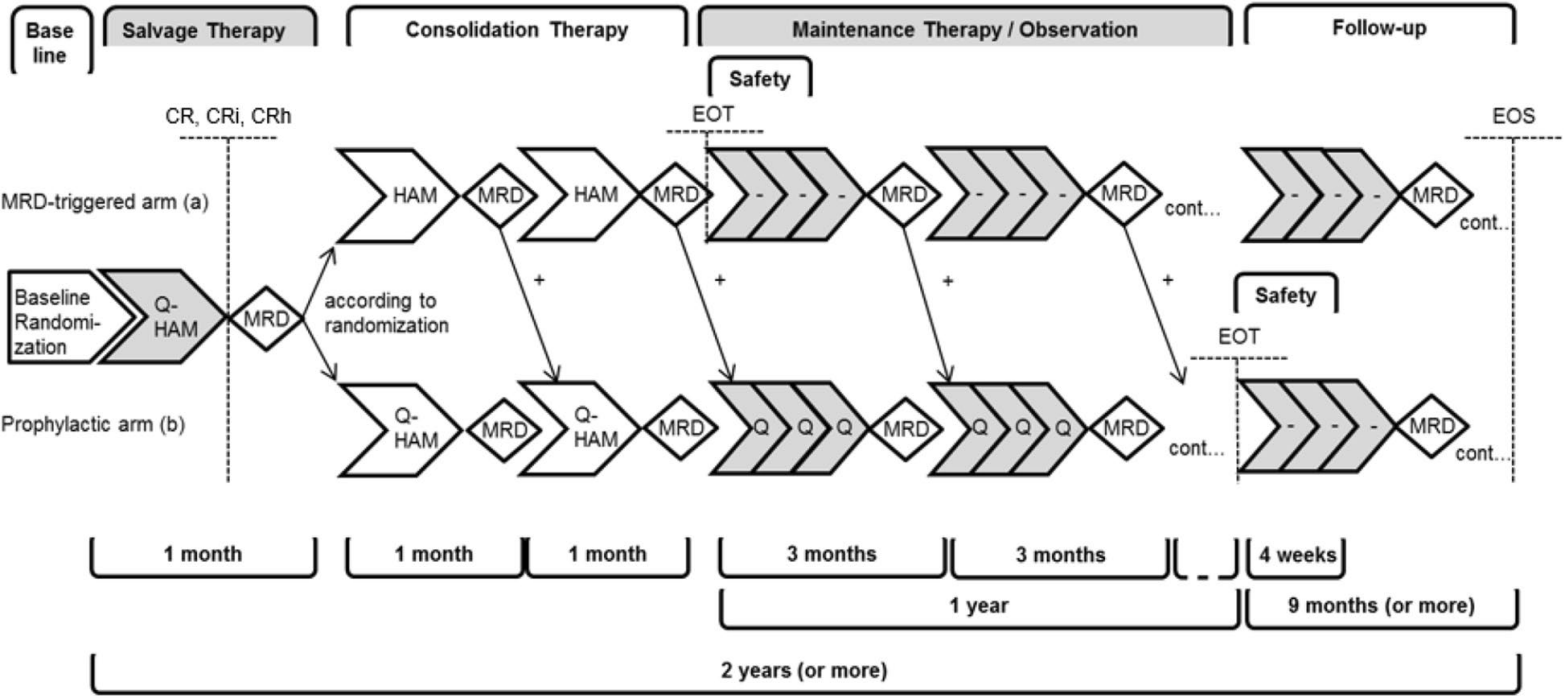
Overall treatment schedule Q-HAM-Study. Abbreviations: MRD, minimal residual disease; CR, complete remission; CRi, complete remission with incomplete hematologic recovery; CRh, complete remission with partial recovery of peripheral blood counts; Q-HAM, quizartinib, high dose cytarabine and mitoxantrone; EOT, end of treatment; EOS, end of study. Please refer to Additional file 1 for a detailed SPIRIT figure

In this multicenter, upfront randomized, open-label, phase II trial, all patients receive quizartinib combined with HAM during salvage therapy. During consolidation therapy (chemotherapy as well as allo-HCT), patients receive either prophylactic quizartinib therapy or MRD-triggered preemptive continuation therapy with quizartinib according to up-front randomization (see Fig. 1 and Additional file 1: SPIRIT figure). Efficacy is assessed by comparison to historical controls based on the matched threshold-crossing approach [10, 25] (Krisam J, Weber D, Schlenk RF, Kieser M: Enhancing single-arm phase II trials by inclusion of matched control patients, unpublished).

### Study setting and randomization

In Germany, patients with r/r-AML are usually referred to academic hospitals. Patients under treatment in the participating centers will be asked to participate in the study. Recruitment will take place in up to 20 academic centers registered in the Study Alliance Leukemia (SAL) group (http://www.sal-aml.org/). During the study conduct, participating centers (see Additional file 2) are contacted by study monitors and the medical coordinator monthly to promote patient recruitment. Furthermore, after protocol amendments or upon relevant updates during the study, a newsletter will be sent to all participating centers. Conferences with the participating centers of the SAL network are held regularly to share information regarding therapy responses and complications seen within the study. Expecting at least 5 potentially eligible patients per year and center, approximately 2 years are required to recruit the anticipated number of patients, when accounting for a consecutive study center initiation and non-participation.

Patients have to provide written informed consent and must meet all inclusion criteria before any protocol-specific procedures are performed.

Each patient must be registered in the electronic case report form (eCRF). During registration, a unique patient ID (PAT-ID) is assigned. Following registration, all eligible patients are upfront 1:1 randomized. The randomization step is triggered by the study sites using a centralized web-based tool (randomizer.at) configured by the responsible data management team. Randomization is performed stratified according to previous treatment with midostaurin. Forty patients are allocated to each arm. Twenty or more centers are selected to recruit the intended number of patients. The treatment arms are (a) the MRD-triggered arm and (b) the prophylactic arm.

### Post-randomization events

In case a patient prematurely withdraws from the trial or is lost to follow-up before measurement of the primary endpoint, this patient’s response is set to missing. In case a patient dies due to any cause before CR/ or CR with incomplete hematological recovery (CRi) or complete remission with partial recovery of peripheral blood counts (CRh) measurement, the primary endpoint will be set to “no Composite Remission”.

### Withdrawal of patients

A patient must be withdrawn from the trial (i) at any time at the patient’s own request, (ii) after salvage therapy if the patient fails to obtain CR/Cri/CRh, (iii) at any time if unacceptable toxicity necessitating cessation of treatment is observed, (iv) at any time if there are changes in the medical status of the patient that compromise the patient’s safety or if the investigator considers that the withdrawal is in the patient’s best interest, (v) in case of pregnancy, (vi) at any time a patient’s protocol compliance turns out insufficient, and (vii) if patient is lost to follow-up.

If any of the aforementioned criteria are met, and patients agree to follow-up, previous unresolved AEs will continuously be followed. If cases iii and iv are met we will continue with the follow-up of the patient until the end of the study. However, if the patient withdraws from the trial and also from consent for disclosure of future information (e.g., follow-up visits), further data collection is prohibited.

### Treatments and study procedures

#### Salvage therapy

All patients are randomized upfront and receive one cycle of backbone salvage therapy with a standard HAM regimen. In patients aged 18–60 years, one cycle of cytarabine 3g/m^2^ every 12 h will be administered intravenously on days one to three, and in patients older than 60 years of age, cytarabine will be administered at a reduced dosage of 1g/m^2^ bidaily on days one to three. Mitoxantrone will be administered irrespective of age group, intravenously at a dosage of 10mg/m^2^ on days two and three. All patients will receive in addition 40 mg of quizartinib orally on days 1 to 28. If on day 28 no hematological recovery has occurred, and according to the first bone marrow examination no CR is achieved, quizartinib may be given until day 42 and a second remission control can be done if medically justified until day 42. If a therapy with a strong CYP3A4 inhibitor is initiated, a dose reduction of quizartinib to 20mg per day will be initiated. Dose modifications in case of toxicities are described in Tables 1 and 2.

**Table 1:** Dose reduction or interruption of quizartinib

Nonhematological events grade 3 related to quizartinib and persisting > 48 h without resolution to ≤ grade 2	Dosing will be interrupted for up to 14 days. If the AE resolves to ≤ grade 1 within 14 days, the subject may resume dosing at the reduced dose of at least 50%.
Grade 4 toxicity at least possibly due to study drug	Treatment will be discontinued
Myelosuppression CRp or CRi	Dose may be reduced without interruption if the following criteria are met: Subject has received a minimum of 2 cycles of ASP2215, platelets < 25 × 10^9^/L and/or ANC ≤ 0.5 × 10^9^/L, marrow blasts < 5%, no evidence of extramedullary disease, further dose reduction is permitted if dosing 1 full cycle at the reduced dose has not resulted in the desired hematologic recovery.

*Abbreviations: AE*, adverse event; *ANC*, absolute neutrophil count; *CRi*, complete remission with incomplete hematologic recovery; *CRp*, complete remission with incomplete platelet recovery

**Table 2:** Dose reduction or interruption of quizartinib in case of QTcF prolongation

QTcF >480 ms ≤500 ms	The dose of quizartinib will be reduced by at least 50%without interruption of dosing. Following dose reduction, the quizartinib dose may be resumed at the previous level in the next cycle if the QTcF has decreased to within 30 ms of baseline or <450 ms.
QTcF >500 ms	Quizartinib dosing will be interrupted for up to 14 days. • If QTcF returns to within 30 ms of baseline or <450 ms within 14 days, quizartinib administration may be resumed at a reduced dose. If QTcF >500 ms occurs during the Induction or Consolidation Phases, and if no cause other than quizartinib can be identified, then during the maintenance phase, the dose of quizartinib cannot be escalated to 60 mg/day.
QTcF >500 ms or >60 ms change from baseline, and Torsade de pointes or polymorphic ventricular tachycardia or signs/symptoms of serious arrhythmia	Quizartinib dosing will be permanently discontinued.

*Abbreviations: QTcF*, corrected QT interval by Fredericia

Subjects who experience QT interval prolongation of more than 480 ms corrected for heart rate (QTcF) will undergo dose interruption and/or reduction of quizartinb and will be monitored closely by ECGs, performed twice weekly for the first week of the QTcF prolongation and then weekly thereafter until the QTcF prolongation is resolved (Table 2). QTcF prolongations higher than grade 3 according to the National Cancer Institute Common Terminology Criteria for Adverse Events (CTC AE) will be recorded as adverse events of special interest (AESI) including the Investigator’s assessment of seriousness, causality, and a detailed narrative.

In the event of significant toxicity grade 3 or 4, dosing must be interrupted, delayed, and/or reduced by at least 50% [20]. In the event of unresolved or severe toxicities, administration will be interrupted and in case of multiple toxicities, dose modification should always be based on the worst toxicity observed. Quizartinib will be discontinued in case of non-hematological grade 4 toxicities including grade 4 QTcF prolongation.

#### Allogeneic hematopoietic cell transplantation

Subjects are permitted to undergo allo-HCT at any time after salvage therapy. Quizartinib should be discontinued at least 7 days before the start of a conditioning regimen. Maintenance therapy may be given starting the earliest at day 30 and the latest at day 100 after transplant according to initial randomization.

#### Continuation therapy

All patients achieving CR are allocated according to randomization to either (a) the MRD-triggered arm or (b) the prophylactic arm receiving treatment with quizartinib. During consolidation therapy, patients allocated to the MRD-triggered arm (a) receive one cycle of HAM and are further allocated based on the MRD results assessed by flow cytometry with a cut of level of 0.1%: if the MRD is negative they remain in the MRD-triggered arm for another HAM cycle, whereas MRD-positive patients move to the prophylactic arm. All patients randomized to (b) the prophylactic arm will receive quizartinib during their two cycles of HAM therapy irrespective of MRD results (see Fig. 1).

Patients achieving MRD negativity in the MRD-triggered arm will not receive treatment during the maintenance phase. MRD-positive patients have to move to the prophylactic arm receiving quizartinib as single agent and will continue the study for up to 12 treatment cycles presenting themselves at monthly visits to assess the course of therapy. Patients in the MRD-triggered arm are attending visits in 3-monthly intervals only. During maintenance therapy, starting from cycle 2, the quizartinib dosage will be increased up to 60mg orally, if the average QTcF of the triplicate ECGs is lower than 450 ms on cycle 1, day 28. Once the dose is increased to 60mg/day, the subject may continue on this dose as long as no dose reduction is needed. In case of therapy with a strong CYP3A4 inhibitor a dose reduction to 20mg per day is required. The maintenance phase is followed by a safety follow-up of 28 days counted from the end of treatment (EOT) for all patients from the prophylactic arm. Dose modifications are listed in Tables 2 and 3.

**Table 3:** Inclusion and exclusion criteria

**Category**	**Inclusion**	**Exclusion**
Population characteristics	- Patients with diagnosed acute myeloid leukemia according to the 2016 WHO classification. who are either • refractory to induction therapy or • relapsed after first line treatment including chemotherapy, autologous and/or allo-HCT* - Positive for FLT3-ITD (defined as a ratio of mutant to wild-type alleles of at least 0.05; measured within 4 weeks before inclusion) - Age ≥ 18 years and ≤ 75 years - ECOG performance status ≤2. - Effective contraception method.	- AML with PML-RARA or BCR-ABL1. - Patients with known active CNS leukemia. - Isolated extramedullary manifestation of AML - Clinically relevant GvHD requiring initiation of treatment or treatment escalation within 21 days prior to screening - Pregnancy and lactation. - Known or suspected active alcohol or drug abuse. - Known positivity for HIV, active HBV, HCV, or hepatitis A infection. - Severe neurologic or psychiatric disorder interfering with ability of giving informed consent. - Hyperleukocytosis (leukocytes > 30,000/μl) at the time of study entry.** - Refractoriness to platelet or packed red cell transfusions
Prior Therapies	- Discontinuation of prior AML treatment for at least • 10 days for cytotoxic agents and • 28 days for investigational drug treatment before the start of study treatment (except hydroxyurea to control hyperleukocytosis)	- allo-HCT within the previous 100 days - Prior treatment with quizartinib
Comorbidities	- Adequate renal function defined as creatinine clearance >50 mL/min (calculated using the standard method for the institution)	- Inadequate renal function. - Inadequate liver function. - Known liver cirrhosis. - History of Sinusoidal Obstruction Syndrome (SOS) - Uncontrolled hypertension. - Severe obstructive restrictive. ventilation disorder. - Myocardial infarction. - Congenital long QT syndrome. - Torsades de pointes. - Arrhythmias (including sustained ventricular tachyarrhythmia). - Right or left bundle branch block and bifascicular block. - Unstable angina. - Coronary/peripheral artery bypass graft. - symptomatic congestive heart failure (NYHA III/IV). - Cerebrovascular accident. - transient ischemic attack. - Symptomatic pulmonary. embolism. - Bradycardia defined as <50 bpms. - QTc interval >470 ms. - Uncontrolled infection. - Evidence or history of severe non-leukemia-associated bleeding diathesis or coagulopathy. - Patients with a “currently active” second malignancy other than non-melanoma skin cancer. - Severe neurologic or psychiatric disorder interfering with ability of giving informed consent.
Others	- Signed written informed consent. - Ability of patient to understand the character and consequences of the clinical trial.	- No consent for biobanking. - History of hypersensitivity to the investigational medicinal product or to any drug with similar chemical structure. - Participation in a clinical study involving an investigational drugs. - No consent for registration, storage, and processing of the individual disease characteristics.

Inclusion and exclusion criteria: The following eligibility criteria are designed to select patients for whom protocol treatment is considered appropriate.* Refractory to induction therapy is defined as no CR, or CRi, or PR (according to standard criteria) [1] after 1 or 2 intensive induction cycles of at least 7 days of cytarabine 100–200mg/m^2^ continuously or an equivalent regimen with cytarabine with total dose not less than 700mg/m^2^ per cycle and 2 days of an anthracycline (e.g., daunorubicin, idarubicin). Relapsed after first-line therapy is defined as relapsed AML (according to standard criteria) [1] after a first-line therapy including at least one intensive induction and consolidation therapy including (but not limited to) allo-HCT. **These patients should be treated with hydroxyurea according to routine practice and are only allowed to enter into the study when leukocyte counts of 30,000/μl or below are reached

*Abbreviations: CNS*, central nervous system; *ECOG*, Eastern Cooperative Oncology Group; *NYHA*, New York Heart Association; *allo-HCT*, allogeneic hematopoietic cell transplantation; *GvHD*, graft-versus-host disease; *AML*, acute myeloid leukemia

#### Long-term follow-up

After EOT, all patients move to the observational follow-up with 3-monthly visits. For patients of the MRD-triggered arm, safety follow-up starts after the last visit of the last cycle of chemotherapy with subsequent transition to observational follow-up until the end of the study (EOS).

After the end of the study visit, patients are routinely followed-up and treated as per the standard of care at the discretion of the treating physician. Follow-up is planned to continue until the last patient alive has been observed for at least 2 years in total (study treatment including subsequent follow-up). Assuming 2 years of recruitment, the total observation period of the first patient may last up to 4 years. Event-free and overall-survival follow-up is recorded until the end of the overall study.

#### Additional study procedures during salvage, maintenance therapy, and follow-up

Patients undergo efficacy and safety assessments, including monitoring of Measurable Residual Disease (MRD), bone marrow specimen collection, and blood and urine sampling before receiving the study drug and at the end of each treatment cycle. During maintenance and follow-up, trial-specific samples are taken every third month. Routine examinations (physical signs, blood values, etc.) are taken at least weekly during treatment. Patient-reported outcome questionnaires are to be completed before treatment and after the end of each treatment cycle, and additionally in 3-month intervals during maintenance and follow-up. AEs are recorded during every visit. Strong CYP3A4 inhibitors should be avoided, and strong or moderate CYP3A4 inducers must not be used.

### Participants

#### Inclusion criteria

Inclusion criteria are outlined in Table 3. Key inclusion criteria are AML according to the 2016 WHO classification, relapse or refractory disease including autologous or allogeneic hematopoietic cell transplantation, positivity for FLT3-ITD mutation defined as a ratio of mutant to wild-type alleles of at least 0.05, age between 18 and 75 years, Eastern Cooperative Oncology Group (ECOG) performance status between 0 and 2, adequate renal and hepatic function, and serum electrolytes (potassium, calcium, and magnesium) within normal limits. Refractoriness to induction therapy is defined as no CR, CRi, or partial remission (PR) after one or two intensive induction cycles of at least 7 days of cytarabine 100–200mg/m^2^ continuously or an equivalent regimen with cytarabine with total dose not less than 700mg/m^2^ per cycle and 2 days of an anthracycline (e.g., daunorubicin, idarubicin). Relapse after first-line therapy is defined as relapsed AML after a first-line therapy including at least one intensive induction and consolidation therapy including allo-HCT. The discontinuation of cytotoxic agents except hydroxyurea to control hyperleukocytosis or investigational treatments for at least 10 days or 28 days respectively is mandatory. Furthermore, all patients randomized into the study must be willing to use highly effective birth control and all female patients must have a negative serum pregnancy test.

#### Exclusion criteria

Exclusion criteria are summarized in Table 3. Main exclusion criteria are diagnosis of acute promyelocytic leukemia (APL) French-American-British classification M3 or World Health Organization classification of APL with translocation t(15;17) (q22;q12), or BCR-ABL–positive leukemia. Other exclusion criteria are known active CNS leukemia, isolated extramedullary manifestation of AML, hyperleukocytosis (leukocytes > 30,000/μl), uncontrolled or significant cardiovascular disease, history of other malignancies (except adequately treated nonmelanoma skin cancer, curatively treated in situ disease, or other solid tumors curatively treated with no evidence of disease for at least 2 years), patients within the first 100 days posterior to allo-HCT, clinically relevant Graft-versus-Host-Disease, QTc interval >450 ms, or any known relevant dysrhythmias.

### Efficacy endpoints

The primary endpoint of the study is the achievement of CR, Cri, or CRh after salvage therapy with HAM in combination with quizartinib. CR is defined as bone marrow blasts <5%, absence of circulating blasts and blasts with Auer rods, absence of extramedullary disease, neutrophil count ≥1000/μl, and platelet count ≥100,000/μl. CRi is defined as CR with residual neutropenia (neutrophils < 1000/μl) or thrombocytopenia (platelets < 100 000/μl) and CRh is defined as a CR with partial recovery of peripheral blood counts with residual neutropenia (neutrophils < 500/μl) or thrombocytopenia (platelets < 50,000/μl).

Refractory disease or treatment failure is defined as failure to achieve CR, Cri, or CRh, presence of Auer rods, or appearance of new or worsening extramedullary disease after one course of intensive treatment. Relapse after CR, CRi, or CRh is characterized by ≥5% blasts in the bone marrow aspirate and/or biopsy not attributable to any other cause, the reappearance of leukemic blasts in the peripheral blood, the new appearance of extramedullary leukemia, or presence of Auer rods. Platelet and neutrophil counts for the assessments of CR, CRi, and CRh are delineated based on the 2017 recommendations from the International Working Group commissioned by the European Leukemia Net [26].

Secondary survival endpoints are event-free survival (EFS), defined from randomization until one of the following events, whichever occurs first: (a) failure to obtain CR or CRi or CRh after Q-HAM therapy, (b) relapse from CR/Cri/CRh, or (c) death from any cause. Patients without an event are censored at the last follow-up. Overall survival (OS) is defined as the time from randomization until death from any cause and relapse-free survival (RFS) is measured from the first CR/Cri/CRh to the time of recurrence of the disease or death from any cause, whichever occurs first. Patients without event are censored at the last date of follow-up. Cumulative incidence of relapse (CIR) is defined as the time from the achievement of a CR/CRi/CRh after salvage therapy to the recurrence of the disease, whereby death from any cause is a competing event. Cumulative incidence of death (CID) is defined as the time from the achievement of a CR/CRi/CRh after salvage therapy to death from any cause whereby recurrence of the disease is a competing event.

Further secondary endpoints are patient-reported outcomes (PROs). PROs include assessments of (a) health-related quality of life (QoL), calculated as the EORTC QLQ-C30 Summary Score [27]; (b) the quality of sleep or sleep disorders, calculated with the “Sleep Quality Index” from the PSQI according to the corresponding scoring guidelines [28]; and (c) anxiety and depression, calculated from the PHQ-4 according to the corresponding scoring manual [29].

### Safety assessments

All adverse events (AEs) that occur after the screening visit (or as soon as the medical history of the patient has been examined) are documented. The period of observation ends with the last study visit. All patients who have AEs, whether considered associated with the use of the investigational medical products or not, are monitored for outcome determination. AEs are collected systematically based on patients’ answers to applicable questions by the investigator, but spontaneous reports are recorded as well if AE criteria apply. For analyses, AEs are centrally coded according to MedDRA. The Data Monitoring Committee (DMC) is composed of three independent experts, meets regularly to review all data relevant to safety, and provides the sponsor with recommendations regarding trial modification, continuation, or termination.

### Auditing

Audits are planned to be performed based on regular risk-based evaluation. Regulatory authorities and auditors authorized by the sponsor may request access to all source documents, the CRF, and other trial documentation. Investigators are contractually bound to enable direct access to these documents and to support audit activities.

### Protocol amendments

Decisions regarding protocol amendments will be taken by the study core team encompassing the coordinating investigator, trial coordinator, trial statistician, medical coordinator, and data management. Meetings for reviewing all available findings and information are scheduled every 2 weeks.

### Sample collection

The samples will be collected at the following time points: (i) at baseline, (ii) after salvage therapy, (iii) after consolidation therapy, (iv) after each cycle of consolidation therapy, (v) after each cycle of maintenance therapy, (vi) at the end of treatment, (vii) every 3 months during observational follow-up, and at end of study. Provided patients’ applicable informed consent is given, biological samples are stored to develop further knowledge and understanding of diagnostic, prognostic and predictive markers present in AML patients.

### Data collection and handling

All data required as per the study protocol, including clinical and laboratory data, are documented by the investigator or an authorized member of the study team in the medical record of the patient and in the eCRF. Access to the eCRF is password protected and an audit trail is in place. Any entries are tracked and locked to prevent further editing. The investigator at the clinical site is responsible for ensuring that all sections of the eCRF are completed correctly. Entries are checked for plausibility and consistency via eCRF-inherent edit checks and visually by the monitors where necessary. Implausibility and missing entries are queried and to be clarified with the responsible investigator. All relevant documents and data collected within the study will be archived for at least 10 years after termination of the study.

### Ancillary and post-trial care

For all patients in the prophylactic arm, treatment ends after the 12th cycle of maintenance therapy (EOT). For all patients randomized to the MRD-triggered arm, who did not cross-over in the prophylactic arm, treatment ends after the 2nd cycle of consolidation therapy (EOT). After EOT patients are routinely followed-up and treated as per the standard of care at the discretion of the treating physician. The period of observation (and the overall study) ends for all patients when the last patient being included and alive has been followed for at least 730 days (2 years) counted from this patient’s day 1 (EOS).

### Study monitoring

Study monitoring is done by the Heidelberg Clinical Studies Coordination Center (KKS). The first monitoring visit at each study center is scheduled to occur at the end of the second patient’s induction therapy. Further monitoring visits at each study site will depend on the (i) recruitment success of study participants, (ii) the monitor’s assessment of the trial site’s compliance with applicable stipulations (e.g., number and severity of protocol deviations or deficiencies detected during study visits), (iii) the deficiencies detected via Central Data Review, and (iv) the assessment of the coordinating team. The monitoring is carried out according to a monitoring manual giving comprehensive guidance on monitoring activities (Source Data Verification rules, corrective and preventive actions, documentation of protocol violations, escalation of findings, etc.).

### Ethical and legal aspects

All the procedures set out in the trial protocol are designed to ensure that all persons involved in the trial abide by Good Clinical Practice (GCP) and the ethical principles described in the current version of the Declaration of Helsinki. The trial is carried out in keeping with local legal and regulatory requirements. The Coordinating Investigator ensures that all trial personnel are adequately trained and maintains a list to whom he has delegated significant trial-related duties.

Before being admitted to the clinical trial, all patients must consent in writing to their participation after having understood the nature, scope, and possible consequences of the clinical trial. Patients are requested to consent to biobanking and to secondary use of their pseudonymized data for yet not determined further scientific analyses. The data obtained in the course of the trial are treated pursuant to the General Data Protection Regulation (EU-DSGVO, EU 2016/679) and the Data Protection Law of the Federal State (Landesdatenschutzgesetz), and the § 40 (2a) AMG. For protection of these data, organizational procedures are implemented to prevent the distribution of data to unauthorized persons.

All planned substantial changes to the study (protocol amendments) are to be submitted to the EC and the competent federal authority requesting their approval. Records of relevant communication with the EC and the regulatory authorities are kept by the coordinating investigator.

### Access to data and dissemination policy

After the publication of the complete trial, access to selected raw data is intended. This must be done in accordance with the European data protection act and informed consent given by the patients.

The results from this trial will be presented at national (e.g., annual meeting of the German Society of Hematology/Oncology); meetings of the Competence Net “Acute and Chronic Leukemias”; and international meetings (e.g., meetings of the European Leukemia-Net; annual congresses of the European Hematology Association, the American Society of Hematology and the American Society of Clinical Oncology). The full results will be published in high-impact peer-reviewed medical journals in accordance with ICMJE guidelines [30]. It is planned to make trial data available for re- and meta-analyses, after the trial is completed and results are published.

### Sample size calculation and statistics

To assess efficacy with respect to the primary endpoint CR/Cri/CRh of Q-HAM during salvage therapy, we will compare the patients in the study to historical controls from previous clinical trials based on the matched threshold-crossing approach [10, 25] (Krisam J, Weber D, Schlenk RF, Kieser M: Enhancing single-arm phase II trials by inclusion of matched control patients, unpublished). Matched controls are drawn from datasets available from individual participant data (IPD) meta-analyses as previously published [6, 7]. The matched threshold-crossing approach is a novel approach to enhance the classic single-arm trial design by including matched historical control patients [31]. This design overcomes common disadvantages of single-armed and small randomized studies [32], since the expected outcome of the observed study population can be adjusted based on the matched controls with a comparable distribution of known prognostic and predictive factors. Furthermore, balanced treatment groups lead to stable statistical models. The proposed adaptive design encompasses two stages with an interim analysis after the first stage [31, 32]. At the interim analysis, historical control patients are matched to the enrolled intervention patients with regard to known prognostic and predictive factors. The treatment effect of the intervention as compared to the control group is then estimated based on the enrolled intervention patients and the selected historical control patients. Results of the interim analysis will be presented to the Data Monitoring Committee (DMC) that will advise the Steering Committee of the trial either to terminate or to continue the trial. In case the estimated treatment effect is below the pre-specified threshold of OR_stop_=1.3, the trial will be stopped for futility. However, in case the estimated treatment effect is above the pre-specified threshold, the sample size for the second stage of the trial will be recalculated to obtain a sufficiently high conditional power based on the results of the interim analysis and the trial continues to the second stage with the recalculated sample size.

In the proposed design, matched historical control patients from two large IPD meta-analyses datasets of r/r-AML patients will be matched by age, and high-risk cytogenetics in refractory patients. Additionally, first CR duration shorter than 18 months will be used as matching factor in relapsed patients. After the enrolment of 20 patients, the a priori unknown matching rate and the number of matched controls per patient in the intervention group are determined by the interim analysis using an iterative procedure (Krisam J, Weber D, Schlenk RF, Kieser M: Enhancing single-arm phase II trials by inclusion of matched control patients, unpublished). An (non-binding) interim stop for futility is done in case the odds ratio, estimated using a logistic regression model adjusting for the matching parameters, does not exceed a threshold of 1.3. Fitting a logistic regression model on both the trial patients and matching historical controls allows to estimate a treatment effect for the novel intervention as compared to standard treatment [32].

If the trial continues to a second stage, a sample size recalculation is performed using a conditional power argument taking the number of matched partners and observed treatment effect into account. In the final analysis, historical control patients are also matched to the patients from the second stage, and the *p*-values from the two stages are combined using the inverse normal approach and tested at a one-sided significance level of *α*=0.05.

The sample size required for a fixed design, assuming CR/CRi/CRh rates of 0.55 for the enrolled patients and 0.30 for the historical controls, would be a minimum of 60 assuming a logistic regression is performed at one-sided significance level *α*=0.05, the aspired power is 0.8, and number of matching partners per intervention patient is at least 1. Using this sample size as a starting point for several simulation studies, it was assessed that the maximal enrolment of 80 patients generated a desirable power of the used adaptive design for most scenarios. The trial sample size is recalculated based on a conditional power argument in an interim analysis after enrolling 20 patients and will hence range between 20 and 80. The assumed CR/CRi/CRh rate of 0.3 for historical controls was estimated based on two large IPD meta-analyses datasets of r/r-AML patients (“historical control”) [6, 7], while the assumed CR/Cri/CRh rate of 0.55 for the patients treated quizartinib was determined to reflect a treatment effect that can be considered as clinically relevant. The sample size for the fixed design was calculated using the software PASS v16, while the simulations we used to evaluate the adaptive design were done using R v3 (http://r-project.org). Randomized patients who do not receive the IMP quizartinib will be replaced. All patients receiving at least one dose of the IMP quizartinib must not be replaced. Due to the underlying disease, loss to follow-up is expected to be negligible.

For the analysis of the primary endpoint, a logistic regression model will be applied to assess the odds ratio of CR/CRi/CRh rate after salvage therapy for patients receiving Q-HAM versus matched controls not receiving Q-HAM. The null hypothesis H0: OR≤1 is tested against its alternative H1: OR>1 at a one-sided significance level of *α*=0.05. The logistic regression analysis will be adjusted for age, high-risk cytogenetics (yes/no), and CR1 duration <18 months (yes/no/not applicable due to refractory disease). Due to the adaptive nature of the trial, two separate logistic models will be fitted for the two trial stages and be combined using the inverse normal function approach. Determination of *p*-values, point, and interval estimates will take the adaptive nature of the trial into account. Concerning the secondary endpoints: EFS, OS, and RFS endpoints will be analyzed using a Cox regression model and Kaplan-Meier plots, CIR, and CID will be analyzed according to Gray 1988 [33] and via cumulative incidence plots, and quality of life will be analyzed descriptively according to the corresponding guidelines and EORTC recommendations. Safety endpoints will be analyzed descriptively. Analyses of the primary and secondary endpoints will be based on the full analysis population (all randomized patients with treatment groups assigned in accordance with the randomization, regardless of the treatment actually received (intention to treat (ITT)). For patients with incomplete follow-up, time to last follow-up date will be used as the censoring time in the analysis of time-to-event data. Otherwise, no imputation of missing data will be conducted. For the biometricians, patient treatment shall remain blinded from the time of randomization until the final database lock. The interim analysis and sample size recalculation will be conducted by an independent biometrician who is not involved in the conduct of the trial. Statistical analysis will be performed using SAS v9.4 or higher [34].

## Discussion

According to one clinical study, patients with *FLT3*-ITD mutated acute myeloid leukemia who relapsed less than 6 months after initial treatment have a dismal prognosis with median overall survival of fewer than four months [35]. Midostaurin plus chemotherapy showed to be efficacious in patients with newly diagnosed *FLT3* mutated AML [11]; however, midostaurin has negligible activity in patients with relapsed or refractory AML. Gilteritinib and quizartinib are possible therapy alternatives in *FLT3*-mutated r/r AML.

Gilteritinib is an oral FLT3/AXL inhibitor, which has been evaluated in a single-agent phase-I/II study [36]. The randomized clinical trial ADMIRAL randomized 371 patients with *FLT3* mutated AML refractory to one or two cycles of conventional anthracycline-containing induction therapy or with hematologic relapse after complete remission. Two hundred forty-seven patients were randomly assigned to gilteritinib single agent and 124 to salvage chemotherapy [37]. Overall (0.64; 95% confidence interval [CI], 0.49 to 0.83; *P*<0.001) and event-free survival (hazard ratio for treatment failure or death, 0.79; 95% CI, 0.58 to 1.09) were better in patients randomized to gilteritinib. Complete remission with full or partial hematologic recovery was achieved in 34.0% in the gilteritinib arm and in only 15.3% in the chemotherapy arm. In addition, a higher proportion of patients in the gilteritinib arm (63 patients, 25.5%) proceeded to allo-HCT compared to the chemotherapy arm (19 patients, 15.3%). However, despite all the beneficial effects of gilteritinib, survival after 24 months was not different between the chemotherapy and gilteritinib arm of the study and was below 20% in both arms.

Quizartinib was evaluated in a phase III randomized, controlled clinical trial (QuANTUM-R), which was conducted in patients with *FLT3-*ITD-positive AML with single-agent quizartinib. In contrast to the ADMIRAL trial, the QuANTUM-R trial included high-risk patients with a duration of first complete remission of ≤6 months and only AML with *FLT3*-ITD. In total, 367 patients were enrolled, of whom 245 were randomly allocated to quizartinib and 122 to chemotherapy alone. Overall survival was significantly longer for quizartinib compared to chemotherapy (hazard ratio (HR) 0·76 [95% CI 0·58-0·98; *p*=0·02]). Seventy-eight patients (32%) in the quizartinib arm and 14 patients (11%) in the chemotherapy arm proceeded to allo-HCT [38]. Complete remission with full or partial hematologic recovery was achieved in 48% in the quizartinib arm and in only 27% in the chemotherapy arm. Again, despite all beneficial effects of quizartinib, survival after 24 months was poor and not different between both therapy arms.

Thus, both studies showed dismal and similar outcomes beyond 24 months in both the *FLT3*-inhibitor and the chemotherapy arms. This was seen despite a much higher percentage of patients achieving a response and proceeding to an allo-HCT in the *FLT3*-inhibitor arms, indicating the development of secondary resistance.

Currently, there is no commonly accepted standard for salvage chemotherapy treatment [39]. Allogeneic hematopoietic cell transplantation offers the highest chance of cure in this clinical circumstance. Thus, the objective of the salvage therapy is to reduce leukemic burden, achieve the best possible remission, and perform a hemopoietic stem-cell transplantation [2]. However, a poor response to salvage therapy in patients with relapsed or refractory *FLT3*-ITD acute myeloid leukemia often prevents them from being bridged to hemopoietic stem-cell transplantation, and according to a previous publication, the best timing of allogeneic hematopoietic cell transplantation is after salvage chemotherapy if CR is achieved [6, 7]. This is further supported by data of an extended Cox regression model combining the time-dependent covariables response to salvage therapy (*P*<0.0001) and possibility to perform an allo-HCT (*P*<0.0001) [7, 40].

Several studies indicate a very low probability for achieving a second CR with standard intensive salvage therapy in patients exhibiting a *FLT3*-ITD, arguing in this clinical situation for experimental approaches [41, 42]. First studies concerning the combination of quizartinib with chemotherapy demonstrated that quizartinib is well tolerated and provided sufficient clinical benefit when administered as 40mg dose once daily [18]. Thus, the comparison of quizartinib with intensive salvage therapy versus chemotherapy alone appears as a logical consequence in terms of efficacy and safety.

We designed a randomized phase-II study to compare quizartinib as adjunct to intensive salvage therapy to salvage therapy alone in terms of CR/CRi/CRh achievement using a threshold crossing approach with matched historical controls based on individual participant data from available datasets [6, 7]. In an attempt to keep sample size on a manageable scale and overcome the common disadvantages of single-armed and small randomized studies, the presented matched-threshold crossing was designed to enhance this single-arm trial by including matched control patients with an equal distribution of known prognostic and predictive factors. Our approach combines matching of historical controls and adaptive sample size recalculation within a two-stage design. Furthermore, it allows us to evaluate the experimental treatment in all included patients, it is more cost-effective than a randomized clinical trial, and it enables us to adjust for known confounders. However, one of the limitations of our study is the inability to adjust for unobserved or unknown confounders.

One of the current challenges in AML treatment is finding effective and well-tolerated post-remission therapies to produce lasting or permanent remissions after achieving a CR since this could lead to a reduction in relapse that is known to be significantly high in this group of patients. Regarding the use and efficacy of maintenance therapy with *FLT3* inhibitors, a recent publication reported the results of the European Group for Blood and Marrow Transplantation (EBMT) registry-based study, which included 462 patients with *FLT3* mutated AML. Sixty-two patients received posttransplant sorafenib: 19 prophylactic, 9 as preemptive therapy, and 34 as a treatment for relapse [43]. The multivariate analysis showed that maintenance with sorafenib significantly improved relapse-free survival (HR= 0.44 [95% CI 0.26–0.75; *p*=0.001). A preplanned pair-matched analysis was performed on data from 26 patients in the sorafenib maintenance group and 26 controls. After a median follow-up of 39 months, the 2-year leukemia-free survival (LFS) and OS were 79% and 83% in the sorafenib group, and 54% and 62% in the control group (*p*=0.002 and 0.007, respectively) [42].

Thus, the second objective of this study is to compare in a randomized manner the efficacy of prophylactic quizartinib therapy to MRD-triggered preemptive continuation therapy with quizartinib in terms of event-free survival. To our knowledge, no study so far has evaluated prophylactic maintenance with quizartinib vs. MRD-triggered continuation quizartinib-therapy.

## Trial status

The first patient was enrolled in October 2020, but patient recruitment was significantly slower than expected. So far 11 patients (13% of the planned sample size) are included in the study. Previously the anticipated number of patients was assumed to be enrolled within approximately 2 years. In the meantime, this turned out not to be feasible without an extension of the recruitment period. As gilteritinib is used increasingly in Germany, we finally decided to close the study prematurely.

### Supplementary Information

**Additional file 1.** 

**Additional file 2.**

**Additional file 3. **Ethical and funding approval documentation.

## Abbreviations

CTCAE: Common Terminology Criteria for Adverse Events
ANC: Absolute neutrophil count
AML: Acute myeloid leukemia
ELN: European LeukemiaNet
CR: Complete remission
CRi: Complete remission with incomplete hematologic recovery
CRc: Composite complete remission
WHO: World Health Organization
SAL: Study Alliance Leukemia
r/r-: Relapsed/refractory
HiDAC: High-dose cytarabine
HAM: High dose with mitoxantrone
ITT: Intention to treat
ICMJE: International Committee of Medical Journal Editors
NCRI: National Cancer Research Institute
AMLSG: German-Austrian Acute Myeloid Leukemia Study Group
MRD: Measurable residual disease
*FLT3*-ITD: Internal tandem duplications of the FMS-related tyrosine kinase 3
TKI: Tyrosine kinase inhibitor
PIA: Plasma inhibitory assay
allo-HCT: Allogeneic hematopoietic cell transplantation
OS: Overall survival
EFS: Event-free survival
ECOG: Eastern Cooperative Oncology Group
ANC: Absolute neutrophil counts
PS: Performance status
IV: Intravenous
CRi: Complete remission with incomplete neutrophil or platelet recovery
CRh: Complete Remission with partial recovery of peripheral blood counts
APL: Acute promyelocytic leukemia
RFS: Relapse-free survival
CIR: Cumulative incidence of relapse
PROs: Patient-reported outcomes
QoL: Health-related quality of life
QTc: Corrected QT interval
AE: Adverse event
DMC: Data Monitoring Committee
RT-qPCR: Real-time quantitative polymerase chain reaction
LSC: Leukemia stem cells
MTD: Maximum tolerated dose
KKS: Clinical Studies Coordination Center
eCRF: Electronic case report form
DMC: Data Monitoring Committee
GCP: Good Clinical Practice
