# Supplementary material for: Q-HAM: a multicenter upfront randomized phase II trial of quizartinib and high-dose Ara-C plus mitoxantrone in relapsed/refractory AML with FLT3-ITD
Source: Trials. 2023 Sep 15;24:591. doi: 10.1186/s13063-023-07421-x (PMC10504729; doi:10.1186/s13063-023-07421-x)
Supplement: Supplementary file 1 — Additional file 1. [file 13063_2023_7421_MOESM1_ESM.docx]

# SPIRIT Figure

| **BL** | Baseline (within 14 days, incl. ST-D1) |
| --- | --- |
| **ST** | Salvage Therapy (1 x 21 days + 21 days optional (42 days in total) recovery period) |
| **EoC** | End of Cycle. Last day including optional recovery period. |
| **CT** | Consolidation Therapy (2 x 28 days + 14 days optional recovery period) |
| **M/O** | Maintenance Therapy (prophylactic arm) / Observation (MRD-triggered arm) (12 cycles).  For MRD-triggered arm only 3-monthly visits. |
| **EOT** | End Of Treatment (Within 7 days after or on Last Visit MT) |
| **SA** | Safety Follow-up (28 days after EOT) |
| **FU** | Observational Follow-up  (3-monthly starting from Last Visit MT until EOS, +/- 7days-window per visit) |
| **EOS** | End of Study (for all patients: 2 years after LPFV) |
| **O** | To be **omitted** if already performed within 48 hours |
| **DA** | To be done **daily.** |
| **W** | To be done **weekly**, preferably at the same day of the week (e.g. the starting day of the therapy). |
| **1st** | Only in **1^st^ FU visit** |
| **2C** | Only in **2^nd^ cycle CT** |
| **3M** | To be done **3-monthly.** |
| **Y** | **After 2 years** counted from day 1, visits on site are not mandatory anymore and may be replaced by contacting the treating physician or mailing the questionnaire. In this case, no further samples will be collected. |
| **[ ]** | optional length in case of needed recovery period |
| SL | Safety lab, values not captured in eCRF |
| **Note** | See chapter ‎7.2 for a **day by day schedule**. **Further details** are given below. |

| **Phase** | **BL** | **ST** | **ST** | **ST** | **ST** | **CT** | **CT** | **CT** | **CT** | **M/O** | **M/O** | **EOT** | **SA** | **FU** | **EOS** |
| --- | --- | --- | --- | --- | --- | --- | --- | --- | --- | --- | --- | --- | --- | --- | --- |
| **Day (of Cycle)** | -14- 0 | 1 | 2-3 | 4-EoC | EoC | 1 | 2-3 | 4-EoC | EoC | 1-27 | 28 |  | 28 |  |  |
| Clinical assessments |  |  |  |  |  |  |  |  |  |  |  |  |  |  |  |
| Signs/ symptoms | X |  |  | x^W^ | X |  |  |  | X |  | x | x^O^ | x | x^3mY^ | x |
| Vital signs | X | x | X^DA^ | x^W^ | x | X^O^ | X^DA^ | x^W^ | x |  | x | X^O^ | x | x^3mY^ | X |
| Physical examination | X | x^O^ |  | x^W^ | x | X^O^ |  | x^W^ | x |  | x | x^O^ | x | x^3mY^ | X |
| ECG | X | X^O^ |  |  |  | X^O^ |  |  |  |  | X | X^O^ | X | x^3mY^ |  |
| Extramedullary involvement | X |  |  |  | X |  |  |  | x |  | x^3M^ | x^O^ |  | x^3mY^ | X |
| PRO | x |  |  |  | X |  |  |  | X^2C^ |  | x^3M^ | X^O^ |  | x^3mY^ | X |
| ECOG PS | X | X^O^ |  |  | x | X^O^ |  |  | x |  | x | x^O^ | x | x^3mY^ | X |
| Laboratory assessments |  |  |  |  |  |  |  |  |  |  |  |  |  |  |  |
| Hematology | X | x^SL^ | X^DA,SL^ | x^W,SL^ | x | X^O,SL^ | X^DA, SL^ | x^W,SL^ | x |  | x | X^O^ | x | x^3mY^ | X |
| Basic blood chemistry | X | X^O,SL^ |  | X^W,SL^ | X | X^O,SL^ |  | X^W,SL^ | X |  | X | X^O^ | X | x^3mY^ | X |
| Ext. b. chemistry & coagulation | X |  |  |  | X |  |  |  | x |  | x | X^O^ | X | x^3mY^ | X |
| Local disease assessment | X |  |  |  | X |  |  |  | X |  | x^3m^ | x^O^ |  | x^3mY^ | x |
| Central laboratory assessments |  |  |  |  |  |  |  |  |  |  |  |  |  |  |  |
| Sample Collection (BM, PB) | X |  |  |  | X |  |  |  | X |  | x^3m^ | X^O^ |  | x^3mY^ | x |
| MRD & Disease status | X |  |  |  | X |  |  |  | X |  | x^3m^ | x^O^ |  | x^3mY^ | x |
| Treatment |  |  |  |  |  |  |  |  |  |  |  |  |  |  |  |
| Quizartinib |  |  |  | x^DA^ |  |  |  | x^DA^ |  | x^DA^ | x |  |  |  |  |
| SOC: HAM |  | x | x^DA^ |  |  | X | X^DA^ |  |  |  |  |  |  |  |  |
| Drug Compliance |  |  |  |  | x |  |  |  | x |  | x | X^O^ |  |  |  |
| Safety |  |  |  |  |  |  |  |  |  |  |  |  |  |  |  |
| Concomitant medications | X | X | X^DA^ | x^W^ | x | X | X^DA^ | x^W^ | X |  | x | X^O^ | X |  |  |
| AE assessment |  | X | X^DA^ | x^W^ | x | X | X^DA^ | x^W^ | X |  | x | X^O^ | X |  |  |
| Pregnancy test (WOCBP only) | X | X^O^ |  |  |  | X |  |  | X^2C^ |  | x^3m^ | X^O^ | X | X^1st^ |  |
| Screening and Baseline |  |  |  |  |  |  |  |  |  |  |  |  |  |  |  |
| Informed consent | X |  |  |  |  |  |  |  |  |  |  |  |  |  |  |
| Demographics & Family History | X |  |  |  |  |  |  |  |  |  |  |  |  |  |  |
| Medical/ oncologic history | X |  |  |  |  |  |  |  |  |  |  |  |  |  |  |
| Genetic Assessment (local) | X |  |  |  |  |  |  |  |  |  |  |  |  |  |  |
| Genetic Assessment (central) | x |  |  |  |  |  |  |  |  |  |  |  |  |  |  |
| Cytogenetics | X |  |  |  |  |  |  |  |  |  |  |  |  |  |  |
| ECHO | X |  |  |  |  |  |  |  |  |  |  |  |  |  |  |
| Abdominal ultrasound | x |  |  |  |  |  |  |  |  |  |  |  |  |  |  |
| Urinalysis | X |  |  |  |  |  |  |  |  |  |  |  |  |  |  |
| Virus diagnostics | X |  |  |  |  |  |  |  |  |  |  |  |  |  |  |
| Enrollment & Randomization | X |  |  |  |  |  |  |  |  |  |  |  |  |  |  |
