# Supplementary material for: Q-HAM: a multicenter upfront randomized phase II trial of quizartinib and high-dose Ara-C plus mitoxantrone in relapsed/refractory AML with FLT3-ITD
Source: Trials. 2023 Sep 15;24:591. doi: 10.1186/s13063-023-07421-x (PMC10504729; doi:10.1186/s13063-023-07421-x)
Supplement: Supplementary file 2 — Additional file 2. [file 13063_2023_7421_MOESM2_ESM.docx]

# List of Q-HAM study centers

1. Department of Internal Medicine V, Heidelberg University Hospital, Heidelberg, Germany.
2. Department of Hematology, Oncology and Palliative Medicine, Community Hospital Bielefeld, Germany.
3. Department of Medicine III, Hospital Chemnitz gGmbH, Germany.
4. Department of Medicine and Polyclinic I, TU Dresden University Hospital, Germany.
5. Department of Medicine IV, Halle (Saale) University Hospital, Germany.
6. Department of Medicine II, Jena University Hospital, Germany.
7. Department of Medicine III, Hospital Karlsruhe, Germany.
8. Department of Hematology, oncology and palliative medicine, Hospital Winnenden, Germany.
9. Department of Medicine II, Frankfurt University Hospital, Germany.
10. Department of Medicine I – Hematology and Cell Therapy, Leipzig University Hospital, Germany.
11. Department of Medicine A, Münster University Hospital, Germany.
12. Department of Inner Medicine V, North Hospital Nürnberg, Germany.
13. Department of Inner Medicine II, Elbland Hospital Riesa, Germany.
14. Department of Medicine II, Diaconal Hospital Schwäbisch-Hall, Germany.
15. Department of Hematology, Oncology and Palliative Medicine, Robert-Bosch Hospital Stuttgart, Germany.
