# Supplementary material for: Q-HAM: a multicenter upfront randomized phase II trial of quizartinib and high-dose Ara-C plus mitoxantrone in relapsed/refractory AML with FLT3-ITD
Source: Trials. 2023 Sep 15;24:591. doi: 10.1186/s13063-023-07421-x (PMC10504729; doi:10.1186/s13063-023-07421-x)
Supplement: Supplementary file 3 — Additional file 3. Ethical and funding approval documentation. [file 13063_2023_7421_MOESM3_ESM.zip › 20191220 ZB 57619 Ersteinreichung EN.pdf]

Non certified translated copy.
2021.11.23

MEDIZINISCHE
FAKULTÄT
HEIDELBERG

Ethics Committee of the Med. HD Alte Glockengießerei 11/1 69115 Heidelberg

NCT Study Center Mr.
Lucian Le Cornet
Im Neuenheimer Feld 130/3
69120 Heidelberg

The evaluation shall be carried out in consultation with the ethics committees involved, also listed in Annex 2.

cc:BfArM by e-mail
Involved ethics committees by e-mail
- Final shipment -

20.12.2019
ts -bh

FAVOURABLE OPINION

EudraCT no:	**2018-002675-17**
Inspection plan code:	**NCT-2017-0545 / HeLeNe-18-03-#545**
Title:	**Quizartinib and High-dose Ara-C plus Mitoxantrone in Relapsed/Refractory AML with FLT3-ITD (Q-HAM)**
Sponsor:	**Ruprecht-Karls-University of Heidelberg Medical Faculty represented in law by Heidelberg University Hospital**
Applicant:	**NCT Study Center, Heidelberg**
LKP:	**Prof. Dr. Richard F. Schlenk, Heidelberg**
Our sign:	**AFmu-576/2019 (Please always specify)**

Dear Sir,

The Ethics Committee considered your above request at its meeting on 26/08/2019. The additional information requested was received on **11/25/2019** and **12/10/2019** and **12/20/2019**, respectively.

The Ethics Committee issues a favourable opinion. Reason: There are no grounds for refusal according to Section 42 (1) AMG. The evaluation is based on the documents listed in Annex 1.

The evaluation shall be valid for the inspection bodies listed in Annex 2, together with their inspectors and alternates.

.

MF
HD

University of Heidelberg Ethics Committee of the Faculty of Medicine

Old bell foundry 11/1 69115 Heidelberg

Phone +49 6221 56264-60 (head office)
Fax +49 6221 56264-80
ethikkommission-I@med.uni-heidelberg.de

www.medizinische-fakultaet-hd.uni- heidelberg.de/ethics-commission

Chair:
Prof. Dr. med. Dr. h.c. Thomas Strowitzki

Vice Chair:
Prof. Dr. med. Klaus Herfarth

Management:
Dr. med. Verena Pfeilschifter

eFeedback
Scan or click

BIC SOLADEST600
IBAN DE64 6005 0101 7421 5004 29

Qualitätsmanagement
DQS
ISO 9001:2015
399893 QM 15

TÜV SÜD
ISO 27001

The positive assessment cannot yet be given for the trial sites listed in Appendix 3, including investigators and alternates, as we have not yet received a final opinion from the responsible ethics committee involved.

The Commissioners listed in Annex 4 took part in the vote. Yours sincerely

Dr. Pfeilschifter, Verena
TeleSec PKS eIDAS QES CA 1
German Telekom AG
Friday, 27. December 2019 15:02 CET

on behalf of

Prof. Dr. med. Dr. h.c. Thomas Strowitzki
Chairman

Attachments
Appendix 1-4

General Notes:

- The ethical and legal responsibility for the conduct of this clinical trial remains with the sponsor, the principal investigator, and the investigators.
- The composition and functioning of the Ethics Committee comply with national laws, regulations and the ICH GCP Guideline as amended from time to time.
- An objection to this Opinion may be raised within one month of publication. The objection must be submitted in writing or recorded at the office of the Ethics Committee of the Medical Faculty Heidelberg, Alte Glockengießerei 11/1, 69115 Heidelberg.
- Aspects of research projects relating to data protection law are only examined by the ethics committee in a cursory manner. This vote/assessment therefore does not replace consultation with the responsible data protection officer. Compliance with the relevant data protection laws and the implementation of the data protection concept are the responsibility of the study director/investigator or sponsor.

Appendix 1

List of documents submitted

Primary Submitted Documents:

Letter dated 05.08.2019
Anl 34_Summary dt, undated
Checklist of the working group of the ethics committees of 02.08.2019
Module 1 of 06.08.2019
Module 2 from 05.08.2019
Confirmation letter EudraCT number dated 04.07.2018
Cost absorption statement dated 15.07.2019.

Versicherungsbestätigungvom08.03.2019,
Versicherungsschein-Nr.: 5701031003018
Contribution invoice dated 08.03.2019, policy number: 81940702 03078 390 2668001 (incl. attachment to invoice).
Asset Register
Patient information and consent form, version _V1_1 of 05.08.2019 Anl 17_Risk_Benefit_Weighing, undated.
Protocol, version 1.2 as of 05.08.2019
INVESTIGATOR'S BROCHURE Quizartinib, version 12.0 as of 04/26/2019
Annex 08_Name and Characterization Active Substances, undated.
CV Prof. Dr. Richard F. Schlenk as at Aug 05, 2019
Certificate "Study Update Hematology and GCP Training" Prof. Dr. Richard F. Schlenk from 11.03.2017 (incl. agenda)
Certificate of Participation "GCP/AMG Training" (Basic Course) Prof. Dr. Richard F. Schlenk from 07.10.2015 (incl. training contents)
Certificate "Study Leader Course" Prof. Dr. Richard F. Schlenk from 23.11.2018 CV Dr. med. Tim Sauer from 05.08.2019
Study Experience Tim Sauer, MD Aug 05, 2019
Certificate "AMG Advanced Course for Examiners / Deputies (Ä2) " Dr. med. Tim Sauer from 02.07.2019
Certificate of attendance "Investigator Course" Dr. med. Tim Sauer from 07.03.2009 (incl. content)
FDS Prof. Dr. Richard F. Schlenk from 07.08.2019 FDS Dr. med. Tim Sauer from 07.08.2019
Site Qualification Form for Examiners, Substitutes (Heidelberg) from 05.08.2019 (P/Deputy)
Centre-specific documents
KKS compulsory consultation notice of 03.07.2019 KKS compulsory consultation notice of 07.08.2019
Contract on the Support of a Clinical Trial (IIT) between Heidelberg University Hospital and Medical Clinic V dated 15.05.2019 (LKP / Medical Director) resp. 27.05.2019 (Commercial Director Uniklinik HD) Responsibility Split dated 27.05.2019 (LKP/IMBI/ QoL DKFZ) resp. 07.06.2019 (KKS Heidelberg) resp. 28.05.2019 (LAB Dept. Internal Medicine V)
Annex 20_Financing, undated
(Sample) contract, submission dated June 12, 2019, undated.
Statement on number of paper-based documents to the participating ethics committees dated 05/08/2019.
Anl 09_Subject of the clinical trial, undated
Annex 10_Number, age and sex of persons concerned, undated Annex 11_Criteria for selection, undated
Annex 12_Justification of gender distribution, undated
Annex 13_Treatment plan, undated
Attachment 15_disclosure of pseudonymised data from 01.08.2019

©EKHD-Zustimmende-Bewertung-als-ff-EK-DV-009-190801

(LKP) Attachment 16_meaning of clinical trial, undated
Annex 25_Methods of investigation envisaged, undated
Annex 26_Simultaneous participation in study, undated

©EKHD-Zustimmende-Bewertung-als-ff-EK-DV-009-190801

Annex 29_Compensation of auditors and compensation of data subjects, undated Annex 30_ Data protection compliance of 15.07.2019 (LKP)
Annex 32_Criteria for suspension or early termination, undated Annex 05_Institutions involved, undated
Annex 33_List of ECs involved, undated
Annex 04_name and address of sponsor, undated CD-ROM

Formal Submission:

Letter from Prof. Richard F. Schlenk dated 08/21/2019 Letter from LKP regarding funding dated 06/28/2019.
Cooperation Agreement between Study Center of the Dept. of Internal Medicine V and Institute of Medical Biometry and Informatics (IMBI) (version of 06/27/2019) dated 06/27/2019 (LKP) and 06/28/2019 (Director IMBI), respectively.
Clinical Trial Agreement between Study Center of the Dept. of Internal Medicine V and Institute of Medical Biometry and Informatics (IMBI) (version of 06/27/2019) dated 06/27/2019 (LKP) and 06/28/2019 (Director IMBI), respectively.
Agreement on the Support of an Investigator Initiated Trial between Heidelberg University Hospital and Daiichi Sankyo Europe GmbH (version of 16.05.2018) dated 27.08.2018 (Kaufmänn Direktorin Uni Heidelberg / Daiichi Sankyo Europe GmbH) resp. 20.08.2018 (LKP) resp. 21.08.2018 (ärztl Direktor Innere Medizin V)
AMENDMENT No. 1 TO Investigator Initiated Study AGREEMENT between Heidelberg University Hospital and Daiichi Sankyo Europe GmbH (version of 14.08.2018) dated 12.09.2018 (Kaufmänn Direktorin Uni Heidelberg) resp. 21.08.2018 (Daiichi Sankyo Europe GmbH) resp. 07.09.2018 (ärztl Direktor Innere Medizin V) resp. 06.09.2018 (LKP)
Sponsor obligations in drug trials in IITs dated 24.06.2019 (LKP)
Budget plan Q-HAM, version dated 02.07.2019.
Annex 00_0_list of facilities, version 01HD dated 21/08/2019
Annex 05_involved facilities dated 21/08/2019.
Annex 33_List of ECs involved as of 15.08.2019
Special agreements for proband annual insurance for universities / university hospitals for clinical trials / studies to be carried out in Germany ("Uni-Jahresvertrag"), as of: undated
General Insurance Conditions for Insurable Clinical Trials of Medicinal Products/Annual Contract (AVB-Prob/AMG-JV) - U 199 -, as at: 1.16
Privacy compliance statement dated 08/22/2019 Your email dated 08/23/2019.

Your email dated 2019-09-02
Confirmation disclosure of pseudonymous data dated 31/08/2019 (LKP).

Your email dated 09/27/2019
Center specific documents.

Your email dated 02/10/2019
Center specific documents.

Letter from Prof. Dr. Schlenk dated 25.11.2019 Centre-specific documents
CD-ROM

Letter from Prof. Dr. Schlenk dated 09.12.2019 List of Annexes
Module 1 from 09.12.2019
Centre-specific documents CD-ROM

©EKHD-Zustimmende-Bewertung-als-ff-EK-DV-009-190801

**Substantive Submission:**	Letter from Prof. Dr. Schlenk dated 25.11.2019 List of Annexes<br>Insurance policy accident insurance no. 50079192101 dated 24.09.2019 General conditions for the SV accident insurance (SVAUB 2017), version January 2017. Supplementary conditions for group accident insurance (ZB Gruppen-UV), version September 2014<br>Data protection information, version 01.10.2018 CD-ROM<br><br>Letter from Prof. Dr. Schlenk dated 09.12.2019 List of Annexes<br>Protocol, version 1.2ek as of 09.12.2019 (with and without changes marked).<br>Patient information and consent form, version 1.1ek as of 09.12.2019 (with and without changes marked).<br>Module 1 from 09.12.2019 CD-ROM<br><br>Letter from Prof. Dr. Schlenk dated 18.12.2019<br>Patient information and consent form, version 1.1ekV2 of 18.12.2019 (with and without marking of the changes)<br>Your email on 12/20/2019

©EKHD-Zustimmende-Bewertung-als-ff-EK-DV-009-190801

Annex 2

The positive evaluation is valid for the following inspection bodies including examiners and deputies:

Ethics Committee	Examiner/Deputy Examiner/Testing Unit
Ethics Committee of the Medical Faculty Heidelberg<br>Old bell foundry 11/1<br>69115 Heidelberg	Prof. Dr. Richard F. Schlenk (P)<br>Dr. med. Tim Sauer (Deputy)<br>Heidelberg University Hospital<br>Dept. of Internal Medicine V<br>Im Neuenheimer Feld 410<br>69120 Heidelberg
Ethics Committee at the TU Dresden<br>Fetscherstraße 74<br>01307 Dresden<br><br>(letter dated Sep. 16, 2019)	PD Dr. med. Christoph Röllig (P)<br>Dr. med. Jan Moritz Middeke (Deputy)<br>University Hospital Carl Gustav Carus Dresden<br>Medical Clinic and Polyclinic I<br>Fetscherstr. 74<br>01307 Dresden
Ethics Committee at the Saxon State Medical Association Schützenhöhe 16<br>01099 Dresden<br><br>(letter, Sept. 20, 2019)	PD Dr. med. Mathias Hänel (P)<br>Dr. med. Regina Herbst<br>(Deputy) Klinikum Chemnitz<br>gGmbH Clinic for Internal<br>Medicine III<br>Haematology/Oncology<br>Bürgerstr. 2<br>09113 Chemnitz
Ethics Committee of the Medical Faculty of the Martin Luther University Halle-Wittenberg<br>Magdeburger Straße 16<br>06112 Halle (Saale)<br><br>(letter dated Sep 04, 2019)	Maxi Wass, MD (P)*<br>PD Dr. med. Lutz Müller (Deputy)<br>University Hospital Halle (Saale)<br>Clinic and Polyclinic for Internal<br>Medicine IV Ernst-Grube-Str. 40<br>06120 Halle (Saale)
Ethics Committee of the Friedrich Schiller University Jena<br>Bachstraße 18<br>07743 Jena<br><br>(letter dated Sep 04, 2019)	PD. Dr. med. Sebastian Scholl (P)<br>Dr. Ulf Schnetzke (Deputy)<br>University Hospital Jena<br>Clinic for Internal Medicine II<br>Haematology and Internal Oncology, Am<br>Klinikum 1<br>07747 Jena
Ethics Committee at the Medical Association of Baden-Württemberg<br>Liebknechtstr. 33<br>70565 Stuttgart<br><br>(letter dated Aug. 28, 2019)<br>(letter dated 08 Oct. 2019)	Dr. med. Thomas Geer (P)<br>Dr. med. Ulrike Sokoll<br>(Deputy) Diakoneo<br>Diakonie Klinikum Schwäbisch Hall gGmbH<br>Internal Medicine III<br>Clinic for Hematology, Oncology and<br>Palliative Medicine<br>Diakoniestrasse 10<br>74523 Schwäbisch Hall
Ethics Committee of the Bavarian Medical Association<br>Mühlbaurstraße 16<br>81677 Munich<br><br>(letter dated Aug. 27, 2019)	Dr. med. Kerstin Schaefer-Eckart (P)<br>Dr. med. Knut Wendelin (Deputy)<br>Paracelsus Medical Private University<br>Nuremberg North Hospital<br>Clinic for Internal Medicine 5 Focus on<br>Oncology / Hematology Prof.-Ernst-<br>Nathan-Str. 1<br>90419 Nuremberg

©EKHD-Zustimmende-Bewertung-als-ff-EK-DV-009-190801

Ethics Committee of the Medical Association of Westphalia-Lippe and the Westphalian Wilhelms University Münster<br>Gartenstr. 210 - 214<br>48147 Münster<br><br>(letter dated Aug. 22, 2019)	PD Dr. med. Martin Görner (P)<br>Dr. med. Kai Wegehenkel<br>(Deputy) Bielefeld Municipal Hospital<br>Clinic for Hematology, Oncology and Palliative Medicine<br>Teutoburger Str. 50<br>33604 Bielefeld

The Ethics Committee involved points out the following:

* Certificate of successful attendance at the advanced course on 27/09/2019 is to be submitted for the examiner in a timely manner.

©EKHD-Zustimmende-Bewertung-als-ff-EK-DV-009-190801

Appendix 9

The positive evaluation cannot yet be given for the following inspection bodies, including examiners and deputies:

Ethics Committee	Examiner/Deputy Examiner/Testing Unit
Ethics Committee at the Saxon State Medical Association Schützenhöhe 16<br>01099 Dresden<br><br>(letter dated Sept. 11, 2019)	Prof. Dr. med. Jörg Schubert (P)<br>Kathrin Petzold (Deputy)<br>Elblandklinikum Riesa<br>Clinic for Internal Medicine II<br>Haematology, oncology, gastroenterology and palliative medicine<br>Vineyard road 8<br>01589 Riesa*
Ethics Committee at the Medical Association of Baden-Württemberg<br>Liebknechtstr. 33<br>70565 Stuttgart<br><br>(letter dated Aug. 28, 2019)	Prof. Dr. med. Mark Ringhoffer (P)**<br>Dr. med. Lukas Kuendgen (Deputy)**<br>Städtisches Klinikum Karlsruhe gGmbH<br>Medical Clinic III<br>Moltke Street 90<br>76133 Karlsruhe
Ethics Committee at the Medical Association of Baden-Württemberg<br>Liebknechtstr. 33<br>70565 Stuttgart<br><br>(letter dated Aug. 28, 2019)	Alexander Reichart, MD (P)<br>Prof. Dr. med. Markus Schaich (Deputy)** Winnenden Hospital<br>Haematology, Oncology and Palliative Medicine Am Jakobsweg 1<br>71364 Winnenden
Ethics Committee of the Medical Association of Westphalia-Lippe and the Westphalian Wilhelms University Münster<br>Gartenstr. 210 - 214<br>48147 Münster	Jan-Henrik Mikesch, MD (P)<br>Prof. Dr. Christoph Schliemann (Deputy) University Hospital Münster<br>Medical Clinic A<br>Hematology, Oncology and Pneumology<br>Albert Schweitzer Campus 1<br>Building A1<br>48149 Münster

The competent ethics committee involved informs the following:

*As per our letter dated 14/08/2019, supplementary documents including qualification certificates have been requested from the applicant and hence it is not yet possible to assess the testing agencies.

The investigators [...] and Dr Ringhofer as well as [...] the deputies Prof. Schaich and Dr Kündgen must provide evidence of an advanced course on clinical trials of medicinal products of at least 8 teaching units of 45 minutes each before the start of the study. The requirements of the German Medical Association of 25.01.2019 must also be taken into account. Evidence of training must be sent to the Commission in a timely manner. Please make sure that the confirmations always contain precise information on the content, time frame and mode of the training (can also be composed of various modules). Proof of participation in a 2-day examiner course that corresponds to the curricular recommendations of the German Medical Association and the Working Group of the Ethics Committees from 2013 can be recognised as equivalent.

©EKHD-Zustimmende-Bewertung-als-ff-EK-DV-009-190801

Appendix 10
The following members participated in the vote:

	Member	Department
☐	Dr. med. Antje Blank<br>Clinical pharmacology	Medic alClinic /Clinical Pharmacology u. Pharmacoepidemiology
☐	Dr. Chobanyan-Jürgens, M.D.<br>Clinical pharmacology	Medic alClinic /Clinical Pharmacology u. Pharmacoepidemiology
☒	Prof. Dr. med. David Czock<br>Clinical pharmacology	Medic alClinic /Clinical Pharmacology u. Pharmacoepidemiology
☐	Prof. Dr. med. Marc Freichel<br>Pharmacology	Pharmacological Institute Heidelberg
☐	Dr. med. Barbara Grün<br>Clinical pharmacology	..ClinicalPharmacology, NationalCenterforTumorDiseases Heidelberg
☐	Prof. Dr. med. Walter E. Haefeli<br>Clinical pharmacology	Medical Clinic /DepartmentClin ical Pharmacology u. Pharmacoepidemiology
☐	Dr. Nicolas Hohmann<br>Pharmacology	FAf.ClinicalPharmacology, NationalCenterforTumorDiseases Heidelberg
☐	Prof. Dr. phil. Michael Anderheiden<br>qualified lawyer	Univ. Heidelberg / Institute for Constitutional Law, Constitutional Theory and Philosophy of Law
☒	Prof. Dr. Jens Bülte<br>qualified lawyer	University of Mannheim / Chair of Criminal Law, Criminal Procedure Law, Economic and Fiscal Criminal Law
☐	Prof. Dr. Ralf Müller-Terpitz<br>qualified lawyer	Univ. Mannheim / Chair of Public Law, Law of Economic Regulation and Media
☐	Prof. Dr. jur. Jochen Taupitz<br>qualified lawyer	InstituteforGerman, EuropeanandInternationalMedicalLaw,HealthLawandBio ethicsof the University of Heidelberg and Mannheim
☒	Prof. Dr. Justo Lorenzo Bermejo<br>Medical biometry	Institute for Medical Biometry and Informatics
☐	Dipl-Stat. Axel Benner<br>Medical biometry	German Cancer Research Center Heidelberg / Dept. of Biostatistics
☐	Dr. sc. hum., Dipl.-Math. Tom Bruckner<br>Medical biometry	Institute for Medical Biometry and Informatics
☐	Prof. Dr. Meinhard Kieser<br>Medical biometry	Institute for Medical Biometry and Informatics
☐	Prof. Dr. Annette Kopp-Schneider<br>Medical biometry	German Cancer Research Center Heidelberg / Dept. of Biostatistics
☐	Dr. sc. hum. Anja Sander, M. Sc<br>Medical biometry	Institute for Medical Biometry and Informatics
☐	Dipl. Math. Svenja Schüler<br>Medical biometry	Institute for Medical Biometry and Informatics
☒	Dr. med. Verena Pfeilschifter<br>Medical Ethics	Office of the Ethics Committee of the Medical Faculty Heidelberg
☐	Michael Reichert<br>Medical Ethics	Help for the elderly of the Evang. Stadtmission Heidelberg
☐	Rebekka Steel<br>Nursing service	Center for Orthopedics, Trauma Surgery and Paraplegiology / Dept. of Orthopedics and Trauma Surgery

©EKHD-Zustimmende-Bewertung-als-ff-EK-DV-009-190801

Appendix

☐	Dr. phil. Nicole Maria Bauer<br>Medical layman	Univ. Innsbruck / Institute for Practical Theology
☐	Christiane Calis<br>Medical layman	Public Relations Office of the City of Heidelberg
☐	Peter Grünewald<br>Medical layman	Theologian, St. Raphael High School Heidelberg
☒	Renate Walter<br>Medical layman	Housewife
☐	Carina Welsch<br>Medical layman	Special Education Teacher

©EKHD-Zustimmende-Bewertung-als-ff-EK-DV-009-190801

☐	Prof. Dr. med. Stefan Delorme<br>Radiology	German Cancer Research Center Heidelberg / Dept. of Radiology
☐	PD Dr. med. Monika Eichinger<br>Radiology	Thorax Clinic Heidelberg / Diagnostic and Interventional Radiology
☐	Dr. med. Sabine Haufe<br>Radiotherapy	Radiology Clinic / Dept. of Nuclear Medicine
☒	Prof. Dr. med. Klaus Herfarth<br>Radiology	Radiology Clinic / Dept. of Radiooncology and Radiotherapy
☐	Angela Paul, MD<br>Radiotherapy	Radiology Clinic / Dept. of Radiooncology and Radiotherapy
☐	Prof. Dr. med. Thomas Strowitzki<br>Gynecology	Gynaecological Clinic/Gynaecological Endocrinology and Fertility disorders
☐	Susanne Picardi, MD<br>Anesthesia	Anaesthesiology Clinic
☐	Prof. Dr. med. Tanja M. Rabsilber<br>Ophthalmology	University Eye Hospital
☐	Prof. Dr. med. Katrin Hoffmann<br>Surgery	Surgical Clinic / Clinic for General, Visceral and Transplant Surgery
☐	Prof. Dr. med. Claudia Sommerer<br>Surgery	Surgical Clinic / Clinic for General, Visceral and Transplant Surgery
☐	Prof. Dr. med. Alexander Scharf<br>Gynecology	Practice for prenatal medicine Darmstadt
☐	PD Dr. med. Johann Greil<br>Pediatrics	Cent. f. Pediatric and Adolescent Medicine / Dept. III Pediatric Hematology and Oncology
☐	PD Dr. med. Peter Ruef<br>Pediatrics	SLK Clinics Heilbronn / Clinic for Paediatrics and Adolescent Medicine
☐	Prof. Dr. med. Claus-Peter Schmitt<br>Pediatrics	Cent. f. Pediatrics and Adolescent Medicine / Dept. of General Pediatrics, Neuropediatrics Metabolism, Gastroenterology, Nephrology
☐	Philipp Ehlermann, MD<br>Cardiology	Medical Clinic / Dept. III Cardiology, Angiology and Pneumology
☐	Dr. med. Jürgen Krauss<br>Oncology	National Center for Tumor Diseases Heidelberg / Dept. of Medical Oncology
☐	Prof. Dr. med. Birgit Friedmann-Bette<br>Sports Medicine	Medical Clinic / Dept. VII Sports Medicine
☐	Dr. med. Dr. med. dent. Julia Katharina Kansy<br>Maxillofacial Surgery	Oral, Dental, Maxillofacial Clinic / Dept. f. Mouth, Jaw & Facial Surgery
☒	Prof. Dr. med. Peter Ringleb<br>Neurology	Neurological Clinic / Dept. of Neuroradiology and Polyclinic
☐	Prof. Dr. med. Thorsten Steiner<br>Neurology	Klinikum Frankfurt Hoechst / Neurological Clinic
☐	Prof. Dr. med. Wolfgang Wick<br>Neurology	Neurological Clinic / Dept. of Neurology and Polyclinic
☐	Prof. Dr. phil. Beate Ditzen<br>Medical psychology	Institute for Medical Psychology
☐	Joanne Nyarangi-Dix, M.D.<br>Urology	Surgical Clinic / Dept. of Urology
☐	PD Dr. Dr. med. Ute Moog<br>Human Genetics	Institute of Human Genetics
