# Supplementary material for: Q-HAM: a multicenter upfront randomized phase II trial of quizartinib and high-dose Ara-C plus mitoxantrone in relapsed/refractory AML with FLT3-ITD
Source: Trials. 2023 Sep 15;24:591. doi: 10.1186/s13063-023-07421-x (PMC10504729; doi:10.1186/s13063-023-07421-x)
Supplement: Supplementary file 3 — Additional file 3. Ethical and funding approval documentation. [file 20180119 IIS Endorsement Letter Prof Schlenk_19_01_2018 geschwärzt.pdf › 20180119 IIS Endorsement Letter Prof Schlenk_19_01_2018 geschwärzt.pdf]

Passion for Innovation.
Compassion for Patients.™

Daiichi-Sankyo

Daiichi Sankyo Europe GmbH · 81366 Munich · Germany

Daiichi Sankyo Europe GmbH
Zielstattstrasse 48
81379 Munich · Germany
Phone +49 89 78080
Fax +49 89 7808267
service@daiichi-sankyo.eu
www.daiichi-sankyo.eu

Prof Dr Richard F. Schlenk
Head of NCT-Trial Center
National Center for Tumor Diseases
Im Neuenheimer Feld 130.3
69120 Heidelberg

Jan 19, 2018

Dear Professor Schlenk,

I am pleased to inform you that the Investigator-Initiated Study (IIS) Committee of Daiichi Sankyo Inc. (DSI) has approved supporting your study entitled **"Quizartinib and High-dose Ara-C plus Mitoxantrone in Relapsed/Refractory AML with FLT3-ITD".**

Please be aware that approval of your study proposals with funding in the total amount of approximately [redacted] is contingent upon review of the final protocols.

After the above is completed, approval will be pending our internal legal office review and preparation of a legal agreement.

The following information will be required by DSE before we can begin working on your legal agreement. Please submit the items indicated below at your earliest convenience.

- ☐ Name, phone, address, and fax number of a contract person in your institution's contract office
- ☐ Bank Account Details

A legal agreement has been drafted by Daiichi Sankyo Europe and will be forwarded to your legal department after the above mentioned information is available.

Please send all of the aforementioned documents via email to [redacted] at [redacted]@daiichi-sankyo.eu. You can reach [redacted] at +49 (0)89 [redacted].

If I can assist you in any way, please feel free to contact me.

HRB 6262 München · Steuer-Nr. 143/127/60445 · UST-ID-Nr. DE 129405556
Geschäftsführer: Martin Hesse, Dr. Jan Van Ruymbeke · Aufsichtsratsvorsitzender: Junichi Fukute
HSBC Trinkaus & Burkhardt AG Düsseldorf 070 0113 019 (BLZ 300 308 80) · Swift TUBDDEDD · IBAN DE18300308800700113019

Passion for Innovation.
Compassion for Patients.™

Daiichi-Sankyo

Sincerely,

Heidi Anthoni, PhD
Daiichi Sankyo Europe
SM Medical Affairs Oncology & Biologics

cc: [redacted], PharmD, Global Medical Affairs Oncology
[redacted] PhD, Clinical Operations
[redacted], MAS, Global Medical Affairs Oncology, Clinical Operations
